# Non-invasive detection of hypovolemia or fluid responsiveness in spontaneously breathing subjects

**DOI:** 10.1186/1471-2253-13-40

**Published:** 2013-11-05

**Authors:** Éva Zöllei, Viktória Bertalan, Andrea Németh, Péter Csábi, Ildikó László, József Kaszaki, László Rudas

**Affiliations:** 1 Department of Anaesthesiology and Intensive Therapy, University of Szeged, Semmelweis u. 6, Szeged 6725, Hungary; 2 Institute of Surgical Research, University of Szeged, Pécsi u. 6, Szeged 6720, Hungary

**Keywords:** Hypovolemia, Functional hemodynamic monitoring, Spontaneous breathing, Microcirculation

## Abstract

**Background:**

In the assessment of hypovolemia the value of functional hemodynamic monitoring during spontaneous breathing is debated. The aim of our study was to investigate in spontaneously breathing subjects the changes in hemodynamic parameters during graded central hypovolemia and to test whether slow patterned breathing improved the discriminative value of stroke volume (SV), pulse pressure (PP), and their variations (SVV, PPV). In addition, we tested the alterations in labial microcirculation.

**Methods:**

20 healthy volunteers participated in our study. Central hypovolemia was induced by lower body negative pressure (LBNP). Continuous signals of ECG, non-invasive blood pressure and central venous pressure were recorded. During baseline and each stage of LBNP the labial microcirculation was investigated by orthogonal polarization spectral imaging, 3 minute periods of patterned breathing at 6 and 15/min respiratory rate were performed, and central venous blood gas analysis was done. Data from baseline and those of different LBNP levels were compared by analysis of variance and those of different breathing rates by t-test. Finally, we performed ROC analysis to assess the discriminative values of SV, PP, SVV and PPV.

**Results:**

Moderate central hypovolemia induced by LBNP caused significant, clinically relevant falls in PP (p < 0.05) and SV and central venous oxygen saturation (ScvO_2_) (p < 0.001). The proportion of perfused vessels (p < 0.001) and microvascular flow index decreased (p < 0.05). PPV increased (p < 0.001), however the magnitude of fluctuations was greater during slow patterned breathing (p < 0.001). SVV increased only during slow patterned breathing (p < 0.001). ROC analysis confirmed the best predictive value for SV (at 56 ml cut-off AUC 0.97, sensitivity 94%, specificity 95%). Slow patterned breathing improved the discriminative value of SVV (p = 0.0023).

**Conclusions:**

Functional hemodynamic monitoring with slow patterned breathing to control spontaneous respiration may be worthy for further study in different populations for the assessment of hypovolemia and the prediction of volume responsiveness.

## Background

The recognition of hypovolemia is of critical importance in the management of our patients. This is especially important in severe civil or military mass trauma situations where a parameter or a combination of parameters would be very useful to help to triage patients who are still “stable” but are approaching cardiovascular collapse due to haemorrhage [1,2]. Furthermore, in Intensive Care Units it is very often necessary to predict if patients with multiple organ failure are hypovolemic, need fluid loading, will tolerate fluid loading and will improve as a result.

Clinical signs and laboratory or blood gas data often help to establish the diagnosis of hypovolemia. Hemodynamic parameters such as cardiac filling pressures (central venous pressure, CVP, and pulmonary artery occlusion pressure, Paop) and static volumetric preload parameters (global end-diastolic volume index, GEDVI; intrathoracic blood volume index, ITBVI) have been used to approximate cardiac preload and to guide fluid therapy.

Recently the so-called dynamic hemodynamic parameters have been found to be more accurate in the diagnosis of hypovolemia and fluid responsiveness [3-5]. For calculating these parameters we use preload alterations (most often induced by respiration) and the resulting changes in either right atrial pressure, blood pressure, pulse pressure, stroke volume or cardiac output. From these fluctuations we can calculate the systolic pressure variation (SPV), pulse pressure variation (PPV) and stroke volume variation (SVV) [5-15]. However, it has been demonstrated in previous studies that these indices are of clinical value only if the patient is mechanically ventilated without spontaneous breathing activity [16-19]. We also know that the level of PEEP, respiratory rate and tidal volume also influence the value of SPV, PPV and SVV [3,9,20-26]. In addition, significant arrhythmias, pulmonary hypertension, increased intraabdominal pressure, right and left heart failure and the use of catecholamines also influence their predictive value [19,27-29].

From investigations on cardiovascular autonomic regulation it is clear that respiration has a fundamental influence on heart rate and blood pressure fluctuations. To control the breathing of awake and cooperative subjects the method of patterned breathing is used. This means that the subjects synchronize inspiration and expiration and sometimes tidal volume to an audio or visual signal. The respiratory frequency can be centred either to the high frequency (respiratory, 0.25 Hz) or low frequency (around 0.1 Hz) bands of cardiovascular fluctuations, which means 15/min and 6/min rate. Doing so, the oscillations in these bands can be enhanced, especially at 6/min breathing rate when the respiratory oscillations and Mayer waves merge.

To induce central hypovolemia in healthy volunteers lower body negative pressure (LBNP) is an accepted, safe and widely used method. This results in the redistribution of blood volume, which generates central hypovolemia without actual volume loss. According to previous data, the volume shift generated by −10-20 mmHg LBNP is similar to mild (400–550 ml), by −20-40 mmHg to moderate (550–1000 ml), and by greater than −40 mmHg to severe (more than 1000 ml) acute blood loss [30].

Experimental and clinical research suggests that in circulatory failure or shock it is the alteration of the microcirculation that finally leads to tissue oxygenation problems and multiple organ failure [31-33]. Furthermore, ultimately the improvement of microcirculatory function determines survival, therefore it is important to know its behaviour during different conditions [32].

The aim of our study was to investigate the effects of mild and moderate LBNP-induced central hypovolemia on the behaviour of hemodynamic parameters, especially on PPV and SVV in spontaneously breathing subjects, and to test the hypothesis that slow patterned breathing improves their discriminative value. Furthermore, we studied the microvascular alteration caused by different levels of central hypovolemia.

## Methods

Our study population consisted of 20 healthy young male volunteers (age 24 ± 6 years), who provided informed consent to participation. Firstly, the subjects were familiarized with the laboratory and equipment, and were trained to perform patterned breathing. In order to control their respiration, patterned breathing was used, so they synchronized inspiration and expiration to an audio signal. Afterwards we inserted a peripherally inserted central line. We ascertained the correct position of the catheter tip by the appearance of the classic central venous pressure (CVP) wave form on the monitor. After this the subjects assumed supine position in the LBNP chamber.

We monitored the ECG, CVP and arterial oxygen saturation continuously using a bedside monitor (Marquette Eagle, Marquette Electronics, Milwaukee, Wi, USA) and arterial blood pressure by the Finapres non-invasive monitor (Ohmeda, Englewood, CO, USA). During the patterned breathing periods we monitored the end tidal CO_2_ level through a mouth-piece connected to a capnograph (Novametrix Medical Systems, Inc., Wallingford, CT, USA) to confirm the subjects’ adherence to the breathing commands. All these signals were digitized by 500 Hz and recorded on-line by the Dataq/Windaq data acquisition system (Dataq Instruments, Inc., Akron, OH, USA).

In order to study microcirculation, we investigated the labial microvessels. For this the orthogonal polarization spectral (OPS) imaging technique was used (Cytoscan A/R, Cytometrix, Philadelphia, PA, USA). A 10× objective was placed onto the inner surface of the labia, and microscopic images were recorded on an S-VHS video recorder (Panasonic AG-TL 700, Matsushita Electric Ind. Co. Ltd, Osaka, Japan).

**Figure 1:**
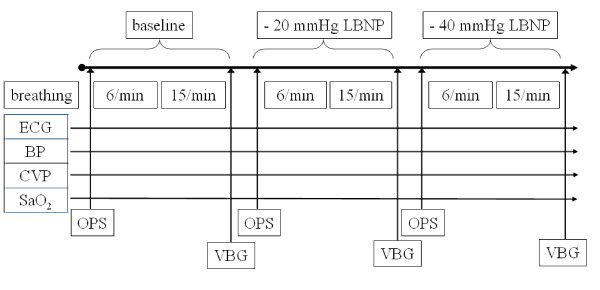
**Study protocol.** (BP: blood pressure, CVP: central venous pressure, SaO_2_: arterial oxygen saturation, OPS: orthogonal polarisation spectral imaging, VBG: central venous blood gas analysis).

Central hypovolemia was induced by −20 and −40 mmHg LBNP. During baseline and each stage of LBNP first the labial microcirculation was investigated and afterwards 3 minute periods of patterned breathing at 6 and 15/min respiratory rate were done. In addition, central venous blood sampling was performed to measure pH, central venous oxygen tension (PcvO_2_), central venous CO_2_ tension (PcvCO_2_), central venous oxygen saturation (ScvO_2_) and haemoglobin (Hb) (Figure 1). The study protocol was approved by the Institutional Human Medical Biological Research Ethics Committee of the University of Szeged, and was carried out in accordance with the Declaration of Helsinki.

For the analysis of hemodynamic data the WinCPRS (Absolute aliens Ay, 2000, Turku, Finland) program was used. For SV calculation this program uses the pulse-contour method (modified Modelflow). First, all the signals were automatically peak-detected and manually checked. Then we calculated RR interval (RRI), systolic arterial pressure (SAP), mean arterial pressure (MAP), pulse pressure (PP) and SV for the entire 3 minute periods in each study condition. Afterwards, we calculated the minimum and maximum RRI, SAP, MAP, CVP, PP and SV for each respiratory cycle during 1 minute of the patterned breathing periods and calculated their differences (delta values) as the respiratory fluctuations (this involved 6 respiratory cycles during 6/min and 15 cycles during 15/min breathing). In addition, we calculated PPV and SVV according to accepted formulas [26].

For the analysis of data from the microcirculation we used a computer-assisted image analysis system (IVM Pictron, Budapest, Hungary). Quantitative assessment of the microcirculatory parameters was performed off-line by frame-to-frame analysis of the videotaped images. We determined red blood cell velocity (RBCV), the mean perfused capillary lengths (MPCL), the proportion of perfused vessels (PRPV) and the microvascular flow index (MFI) in accordance with accepted methods [34]. All microcirculatory evaluations were performed by the same investigator, who was not blinded to the study conditions, but was not aware of the results of the hemodynamic assessment (IL).

Data from baseline and those of different LBNP levels were compared by analysis of variance and those of the two different breathing rates by t-test. In the case of non-normal distribution of data, analysis of variance on ranks and the Mann–Whitney rank sum test were used. (Sigmastat, Aspire Software International, Ashburn, VA, USA). In order to assess the discriminative value of data, ROC analysis was done. SV and PP during 6/min, and SVV and PPV during both respiratory rates were analysed by using data from baseline and −40 mmHg LBNP (Medcalc, Mariakerke, Belgium). Data are given as mean ± SD. P value below 0.05 was considered to be statistically significant.

## Results

**Figure 2:**
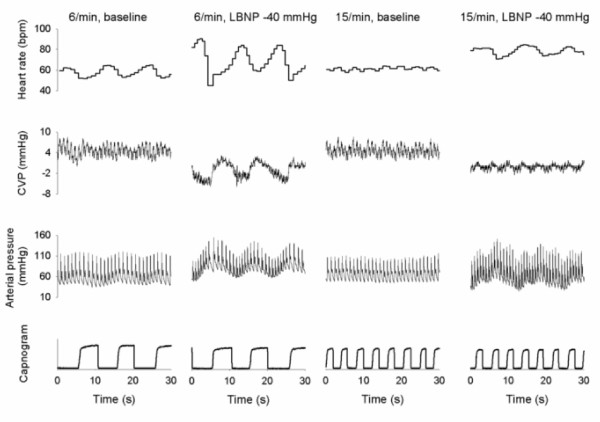
The typical recording of one subject at the different LBNP levels and at 6/min and 15/min patterned breathing.

The typical recording of one subject is shown in Figure 2. at different LBNP levels and at 6/min and 15/min patterned breathing, which demonstrates the enhanced fluctuations at moderate hypovolemia and slow patterned breathing in heart rate, arterial blood pressure and central venous pressure. The whole protocol was completed in 11 subjects. 4 of them had presyncopal symptoms, and −40 mmHg LBNP could not be performed (therefore during moderate central hypovolemia we analysed data from 16 volunteers), and in another 5 PIC line insertion was unsuccessful (as a result we have CVP data from 15 volunteers).

### Hemodynamic, central venous blood gas and microcirculatory parameters at different levels of LBNP

From the hemodynamic parameters MAP did not change; but RRI, SAP, CVP, PP and SV significantly decreased at −40 mmHg, and CVP and SV even at −20 mmHg LBNP. In the case of RRI, SAP, PP and SV there was also a significant difference between −20 and −40 mmHg LBNP (Table 1).

**Table 1:** Hemodynamic parameters, central venous blood gases and microcirculatory variables

	**Baseline**	**−20 mmHg LBNP**	**−40 mmHg LBNP**
RRI (msec)	859 ± 159	844 ± 144	739 ± 115^##§§^
SAP (mmHg)	130 ± 17	126 ± 15	123 ± 11^##§^
MAP (mmHg)	83 ± 13	84 ± 10	87 ± 8
CVP (mmHg)	7.2 ± 2.0	3.7 ± 2.0^**^	2.6 ± 2.0^##^
PP (mmHg)	65 ± 10	58 ± 10	50 ± 7^#§^
SV (ml)	79 ± 12	63 ± 12^**^	50 ± 8^##§§^
pH	7.38 ± 0.02	7.39 ± 0.03	7.39 ± 0.04^#^
pCO_2_ (mmHg)	45 ± 3	43 ± 4	43 ± 5^#^
pO_2_ (mmHg)	42 ± 5	37 ± 4^*^	36 ± 4^##^
ScvO_2_ (%)	76 ± 6	70 ± 7^*^	67 ± 9^##^
Hb (g/dl)	13.7 ± 0.8	14 ± 0.8^**^	14.5 ± 0.9^##§§^
RBCV (microm/s)	1035 ± 67	792 ± 153^**^	696 ± 156^##§^
MPCL (microm)	2696 ± 593	2194 ± 567^**^	1973 ± 512^##^
PRPV (%)	99.7 ± 1.5	90.4 ± 13^*^	87.8 ± 13.9^##^
MFI	3.0 ± 0	2.8 ± 0.4 ^*^	2.7 ± 0.4 ^#^

Hemodynamic data are calculated from the whole 3 minute recordings at 6/min patterned breathing at baseline and during mild (−20 mmHg LBNP) and moderate (−40 mmHg LBNP) central hypovolemia and are given as mean ± SD.

^*^baseline vs. -20 mmHg, p < 0.05.

^**^baseline vs. -20 mmHg, p < 0.001.

^#^baseline vs. -40 mmHg**,** p < 0.05.

^##^baseline vs. -40 mmHg**,** p < 0.001.

^§^-20 vs −40 mmHg, p < 0.05.

^§§^-20 vs −40 mmHg, p < 0.001.

Central venous blood gas analysis showed that pH and Hb significantly increased, pCO_2_, pO_2_ and ScvO_2_ significantly deceased at −40 mmHg LBNP, and this change was even significant between baseline and −20 mmHg in Hb, pO_2_ and ScvO_2_, and between −20 mmHg and −40 mmHg in Hb (Table 1).

All the microcirculatory variables (RBCV, MPCL, PRPV and MFI) decreased significantly between baseline and both LBNP levels, and RBCV between −20 and −40 mmHg LBNP, too (Table 1).

### Respiratory fluctuations at different levels of LBNP and during different respiratory rates

RRI, SAP, MAP, CVP, SV and PP data at different levels of LBNP and at different respiratory rates are shown in Table 2.

**Table 2:** The respiratory fluctuations of hemodynamic parameters

	**6/min patterned breathing**			**15/min patterned breathing**		
	**Baseline**	**−20 mmHg**	**−40 mmHg**	**Baseline**	**−20 mmHg**	**−40 mmHg**
RRI (msec) min	767 ± 130	742 ± 130	651 ± 90^#§^	823 ± 151	793 ± 164	674 ± 127^##§§^
max	952 ± 157	950 ± 177	823 ± 151^##§§^	889 ± 171	848 ± 177^*^	726 ± 145^##§§^
delta	185 ± 86	208 ± 103	173 ± 84	65 ± 32	54 ± 24	52 ± 25
SAP (mmHg) min	123 ± 17	117 ± 16	113 ± 11^#§^	125 ± 14	122 ± 14	118 ± 11^#§^
max	135 ± 18	133 ± 15	131 ± 12^§^	131 ± 15	128 ± 13	125 ± 11^#§^
delta	12 ± 4	16 ± 6^**^	18 ± 8^##^	6 ± 2	6 ± 2	7 ± 3^#^
MAP (mmHg) min	78 ± 12	77 ± 11	78 ± 9	81 ± 11	83 ± 10	86 ± 8
max	87 ± 13	90 ± 10	94 ± 9	85 ± 12	87 ± 10	91 ± 8
delta	9 ± 4	12 ± 5^**^	15 ± 6^##§^	3 ± 1	4 ± 1	5 ± 2^#^
CVP (mmHg) min	2.7 ± 2.4	−0.2 ± 2.3^**^	−0.7 ± 2.5^##^	3 ± 2.7	1.5 ± 2.4^*^	−0.35 ± 2.8^##§^
max	12 ± 3.1	6.7 ± 2.8^**^	5.5 ± 2.6^##^	10 ± 2.8	5.8 ± 2.3^**^	4.6 ± 2.2^##^
delta	9.3 ± 1.8	6.9 ± 1.7^**^	6.2 ± 1.8^##^	7 ± 2.2	4.4 ± 1.5^**^	4.9 ± 1.7^#^
PP (mmHg) min	60 ± 10	52 ± 10^*^	44 ± 5^#§^	60 ± 7	53 ± 8^**^	43 ± 7^##§§^
max	68 ± 11	61 ± 11^**^	53 ± 7^##§§^	64 ± 7	57 ± 8^**^	49 ± 7^##§§^
delta	8 ± 4	9 ± 4	10 ± 4	4 ± 1	4 ± 2	5 ± 2
SV (ml) min	72 ± 11	55 ± 10^**^	42 ± 6^##§§^	70 ± 10	55 ± 9^**^	43 ± 6^##§§^
max	85 ± 13	67 ± 14^**^	55 ± 10^##§§^	79 ± 12	64 ± 11^**^	50 ± 7^##§§^
delta	12 ± 5	13 ± 8	14 ± 8	9 ± 4	8 ± 4	7 ± 3

The data for RRI, SAP, MAP, CVP, PP and SV were calculated from 1 minute recordings (so from 6 and 15 respiratory cycles), during mild and moderate central hypovolemia and at 6/min and 15/min patterned breathing and are given as mean ± SD.

^*^baseline vs. -20 mmHg, p < 0.05.

^**^baseline vs. -20 mmHg, p < 0.001.

^#^baseline vs. -40 mmHg**,** p < 0.05.

^##^baseline vs. -40 mmHg**,** p < 0.001.

^§^-20 vs −40 mmHg, p < 0.05.

^§§^-20 vs −40 mmHg, p < 0.001.

Delta RRIs were not influenced by the LBNP level, but their values were significantly greater during 6/min patterned breathing at all LBNP levels (p<0.001).

SAP and MAP respiratory fluctuations increased at both 6/min and 15/min patterned breathing in moderate central hypovolemia, but in mild central hypovolemia these changes were only significant at 6/min breathing rate. The SAP respiratory fluctuations were greater at slow patterned breathing at each LBNP level (p<0.001). MAP delta increased between −20 and −40 mmHg LBNP only at 6/min patterned breathing.

The CVP delta values changed significantly between baseline and mild and moderate central hypovolemia, but the fluctuations became smaller at both 6 and 15/min respiratory rate.

The assessment of SV, PP, SVV and PPV gave the most interesting results. The minimum, and maximum values of SV and PP decreased in response to all levels of hypovolemia, though the delta values did not change either at 6/min nor at 15/min breathing rate. The fluctuations of both SV and PP were more pronounced at 6/min patterned breathing, and the differences were significant in all comparisons (p<0.001 for PP; p<0.05 for SV), except at baseline in SV. However, PPV increased from baseline to −40 mmHg LBNP, especially at slow patterned breathing. PPV increased also between −20 and −40 mmHg LBNP at 15/min patterned breathing. SVV only increased at slow patterned breathing in moderate central hypovolemia, though it was greater in all 3 conditions with slow respiratory rate (Table 3).

**Table 3:** PPV and SVV during different levels of central hypovolemia and different respiratory rates

	**Baseline**	**−20 mmHg LBNP**	**−40 mmHg LBNP**
PPV 6 (%)	13 ± 5	16 ± 7	20 ± 8^##^
PPV 15 (%)	7 ± 3^**^	8 ± 4^**^	11 ± 4^##§**^
SVV 6 (%)	15 ± 5	20 ± 10	26 ± 12^##^
SVV 15 (%)	12 ± 4^*^	13 ± 5^*^	15 ± 6^*^

Data are calculated from 1 minute recordings (so from 6 and 15 respiratory cycles) at 6/min (PPV 6, SVV 6) and 15/min (PPV 15, SVV 15) patterned breathing and are given as mean ± SD.

^##^baseline vs. -40 mmHg**,** p < 0.001.

^§^-20 vs −40 mmHg, p < 0.05.

^*^6 vs 15/min patterned breathing, p < 0.05.

^**^6 vs 15/min patterned breathing, p < 0.001.

Finally we performed ROC analysis (Table 4). The AUC was the largest and the sensitivity and specificity the best for SV with a 56 ml cut-off, though PP also had acceptable sensitivity and specificity. Slow patterned breathing improved the discriminative value of SVV.

**Table 4:** ROC analysis of SV, PP, SVV and PPV

	**AUC (CI)**	**Cut-off**	**Sensitivity (%)**	**Specificity (%)**
SV	0.97 (0.85-1.0)	56 ml	94	95
PP	0.88 (0,73-0.97)	53 mmHg	88	85
SVV 6	0.83 (0.67-0.94)	16%	81	75
PPV 6	0.78 (0.61-0.9)	13%	81	65
SVV 15^*^	0.69 (0.51-0.83)	13%	69	70
PPV 15	0.83 (0.66-0.93)	8%	81	75

Data calculated from 6/min (SV, PP, SVV 6, PPV 6) and 15/min (PPV 15, SVV 15) patterned breathing during −40 mmHg LBNP.

^*^6 vs 15/min patterned breathing, p < 0.0023.

## Discussion

The main findings of our study are as follows: mild and moderate central hypovolemia induced by LBNP caused significant and clinically relevant falls in PP and SV. This was accompanied by significant and relevant decreases in PcvO_2_ and ScvO_2_. All the investigated microcirculatory variables such as RBCV, MPCL, PRPV and MFI decreased significantly at both levels of central hypovolemia, which indicates progressive impairment of both microvessel density and perfusion. PPV increased at both 6/min and 15/min patterned breathing in moderate central hypovolemia however the magnitude of fluctuations was greater at the slow respiratory rate. SVV increased only during slow breathing, and its accentuation with low respiratory rate was less pronounced then that of PPV. To our knowledge this is the first study that investigates SVV and PPV during LBNP and at different rates of patterned breathing. ROC analysis confirmed the best predictive value for SV absolute values in this group of healthy volunteers. Slow patterned breathing improved the discriminative value of SVV, but not that of PPV.

Recently the effects of LBNP induced hypovolemia on systemic and tissue oxygenation and microcirculation have been investigated in two studies, in which sublingual side stream dark field imaging has been used in healthy volunteers [35,36]. In both they increased the LBNP level until hemodynamic decompensation occurred. Ward et al. found significant decrease in systemic oxygen delivery accompanied by progressive decrease in sublingual functional capillary density (FCD). The oxygen extraction ratio increased both at systemic and local (muscle) level. The fall of FCD occurred earlier than any other change in tissue oxygenation parameters. Bartels et al. found significant decrease not only in perfused vessel density but also in the microvascular flow index, as well as a significant increase in microvascular flow heterogeneity. Our results regarding the microcirculatory alterations were similar, confirming that even −20 mmHg LBNP causes hemodynamic changes great enough to influence microcirculation. The fall in ScvO2 with increasing levels of LBNP is consistent with decreased cardiac output and oxygen delivery, if we assume that oxygen consumption did not change during supine rest.

Since there is strong clinical need to recognize hypovolemia in patients who are not mechanically ventilated, we performed our investigations in spontaneously breathing subjects. The mechanism of respiratory fluctuations in stroke volume and blood pressure is different during spontaneous breathing and positive pressure ventilation [10,11,15,37]. During mechanical ventilation positive pressure inspiration impedes venous return and can increase right ventricular (RV) afterload. These result in a decrease of left ventricular (LV) preload a few heart beats later. However, inspiration also squeezes blood from the pulmonary capillaries towards the left ventricle, and at the same time decreases LV afterload by decreasing aortic transmural pressure. These effects result in an early inspiratory increase in LV SV and arterial pressure, which is called delta up. This is followed by an inspiratory decrease a few heart beats later, which is called delta down. During expiration the RV preload increases, the afterload decreases, therefore RV SV increases. In the left side of the heart however the effects are the opposite, the preload decreases, the afterload increases, which results in lower LV SV and blood pressure. This is followed by an increase in SV and SAP a few beats later when the increased RV SV reaches the left side of the heart. In this way the delta up results mainly from the increase in LV preload and reduction of its afterload, and delta down mainly from decreased venous return to the right heart.

During spontaneous breathing the mechanism of stroke output changes is different. During spontaneous inspiration the venous return increases without a considerable increase in RV afterload. On the left side there is no afterload reduction, so the inspiratory increase in LV SV and arterial pressure (delta up) is the result of the increased RV output. During exhalation, the venous return and RV SV decrease, which reaches the LV a few beats later. At the same time the aortic transmural pressure decreases compared to the inspiratory value, which augments LV ejection. This dampens the effect of decreased preload, so delta down is not so prominent, as during positive pressure ventilation. In summary, the mechanism of delta up and delta down is different: during mechanical ventilation it is the delta down, during spontaneous breathing it is the delta up that is the volume sensitive, preload determined variable.

There is another problem with dynamic hemodynamic parameters besides the above discussed issues. The respiratory perturbation should be great enough to cause significant preload alteration in order for clinicians to be able to assess its effects on blood pressure and left ventricular stroke volume. These effects are smaller during quiet spontaneous breathing and are clearly tidal volume dependent during mechanical ventilation [6,16-18,20-22,24,25].

In addition, the lag between the changes of RV and LV output is determined by the pulmonary transit time. The timing of the influence of RV output changes on the LV is clearly dependent on the heart rate (HR) and respiratory rate (RR) as well as their timing to each other. This relationship is highlighted in the work of De Backer et al. [23]. They concluded that the respiratory variations in left but not in RV stroke volume and its derivates disappeared with high RR, and when the HR/RR ratio fell below 3.6. This relationship however can change from breath to breath during spontaneous respiration, or when the patient triggers the ventilator.

Based on these findings it may be assumed that there is a need to standardize conditions, especially respiratory stimuli when assessing preload responsiveness by dynamic parameters. Perel et al. tried to achieve this with a ventilatory manoeuvre called respiratory systemic variation test (RSVT) [38]. In our study we tried to control the spontaneous breathing of our subject by patterned breathing. The 6/min respiratory rate looked more promising because it merges the effects of sympathetic activity (Mayer waves) and respiration thus increasing blood pressure fluctuations. Besides this, during slow patterned breathing the tidal volume is greater than during quiet breathing, because the subjects automatically adjust it to keep minute ventilation constant. We hypothesized that this stimulus would be regular and great enough to cause measurable changes in dynamic parameters and improve their discriminative value compared to 15/min breathing rate, which served as a control, being similar to normal quite breathing. According to our results, although the fluctuations in blood pressure and SVV and PPV increased, ROC analysis confirmed improvement in discriminative value only of SVV. Still absolute values of SV and PP performed much better in this group of healthy volunteers.

The different behaviour of SVV and PPV may be explained by the fact that the relationship between SV and PP is not linear, arterial distensibility continues to decrease with progressive reduction of central blood volume [1,29].

Finally, we were interested in how the respiratory fluctuations of CVP were influenced, because this is easy to measure and is readily available. In his early study Magder tested the hypothesis that the respiratory pattern of right atrial pressure (RAP) could be used to predict the response to a fluid challenge. He concluded that it was very unlikely that volume loading would increase cardiac output when the inspiratory fall in RAP was less 1 mmHg [13]. Although this fall was greater than 1 mmHg in our subjects even at baseline, the amplitude of fluctuations decreased during mild and moderate hypovolemia. We explain this by the ceiling effect due to the collapse of large veins.

This study has some limitations, the first one being that we investigated healthy young individuals with intact autonomic regulation therefore the results cannot be extrapolated to different patient populations. Another limitation was that our subjects were fully awake and cooperative and after a short learning period could follow the breathing commands properly. The implementation of slow patterned breathing could be more difficult and even impossible in many clinical situations where patients have dyspnea, tachypnea or altered mental state. However we believe that not only during spontaneous but also during controlled mechanical ventilation there is a need to standardise the respiratory pattern when we want to assess functional hemodynamic parameters. For stroke volume analysis we used pulse-contour analysis with modified Modelflow method and verified manually the beat-to-beat automatic wave form analysis. Furthermore, when we investigated the effect of respiratory rate, we averaged data for 1 minute, from 6 and 15 respiratory cycles. Commercially available, built-in systems, with different softwares and time-averaged data could give different results [27].

## Conclusions

In conclusion, slow patterned breathing may allow the assessment of hypovolemia in spontaneously breathing subjects and it is worthy for further study in different conditions and populations. In Emergency Departments and wards patients are usually not equipped with arterial lines and invasive hemodynamic monitoring is not available. In this patient group, for those, who are able to sustain a specific breathing pattern for a few minutes, the use of non-invasive blood pressure monitoring coupled with functional hemodynamic monitoring, is promising.

### Key messages

Mild and moderate central hypovolemia induced by LBNP caused significant and clinically relevant falls in PP and SV. This was accompanied by significant and relevant decreases in PcvO_2_ and ScvO_2_ and progressive impairment of both microvessel density and perfusion.

PPV increased during both 6/min and 15/min patterned breathing in moderate central hypovolemia however the magnitude of fluctuations was greater during the slow respiratory rate. SVV increased only during slow breathing, and its accentuation with low respiratory rate was less pronounced then that of PPV.

ROC analysis confirmed the best predictive value for SV absolute values in this group of healthy volunteers.

Slow patterned breathing improved the discriminative value of SVV, but not that of PPV.

## Abbreviations

AUC: Area under the curve; BP: Blood pressure; CVP: Central venous pressure; ECG: Electrocardiogram; FCD: Functional capillary density; GEDVI: Global end diastolic volume index; Hb: Hemoglobin; HR: Heart rate; ITBVI: Intrathoracic blood volume index; LBNP: Lower body negative pressure; LV: Left ventricle; MAP: Mean arterial pressure; MFI: Microvascular flow index; MPCL: Mean perfused capillary lenght; OPS: Orthogonal polarization spectral imaging; Paop: Pulmonary artery occlusion pressure; PcvO2: Central venous oxygen tension; PcvCO2: Central venous CO_2_ tension; PEEP: Positive end exspiratory pressure; PP: Pulse pressure; PPV: Pulse pressure variation; PRPV: Proportion of perfused vessels; RAP: Right atrial pressure; RBCV: Red blood cell velocity; ROC: Receiver operating characteristic curve; RR: Respiratory rate; RRI: RR interval (the time of the cardiac cycle); RSVT: Respiratory systemic variation test; RV: Right ventricle; SaO2: Arterial oxygen saturation; SAP: Systolic arterial pressure; ScvO2: Central venous oxygen saturation; SV: Stroke volume; SVV: Stroke volume variation; VBG: Central venous blood gas analysis.

## Competing interests

The authors declare that they have no competing interests.

## Authors’ contributions

ÉZ and LR planned the study design, and contributed to hemodynamic data acquisition and analysis. The manuscript was written by ÉZ. VB, AN, PCs have been involved in coordination, hemodynamic data acquisition and analysis. The investigation and data analysis of the microcirculation was done by JK and IL. All authors were involved in the critical revision of the manuscript. All authors read and approved the final manuscript.

## Pre-publication history

The pre-publication history for this paper can be accessed here:

http://www.biomedcentral.com/1471-2253/13/40/prepub
